# Prevalence and associated factors of health facility delivery during COVID-19 in the Tamale Metropolis of Ghana: Analytical cross-sectional study

**DOI:** 10.1371/journal.pone.0344205

**Published:** 2026-03-09

**Authors:** Obed Kwaku Duah Asumadu, Gilbert Abotisem Abiiro, Joyce Aputere Ndago, David Abatanie Kanligi, Martin Nyaaba Adokiya

**Affiliations:** 1 Department of Social and Behavioural Change, School of Public Health, University for Development Studies, Tamale, Ghana; 2 Rehabcare, Patrickswell, Co., Limerick, Ireland; 3 Department of Health Services, Policy, Planning, Management and Economics, School of Public Health, University for Development Studies, Tamale, Ghana; 4 Department of Social and Behavioural Science, School of Public Health, University of Ghana, Legon, Ghana; 5 Pediatric Unit, Savelugu Municipal Hospital, Ghana Health Service, Northern Region, Ghana; 6 Department of Global Health, School of Public Health, University for Development Studies, Tamale, Ghana; Fayetteville State University, UNITED STATES OF AMERICA

## Abstract

**Introduction:**

Globally, the COVID-19 pandemic significantly impacted the provision of maternal health services, especially facility-based delivery. However, there is little evidence on the proportion of women who delivered at the health facility in various locations and the factors that influenced women’s decision-making in choosing a place of delivery during and amid the COVID-19 restrictions. Therefore, this study assessed the prevalence and factors associated with health facility delivery during the COVID-19 pandemic in the Tamale Metropolis of Ghana.

**Methods:**

An analytical cross-sectional study design was conducted. A multistage sampling technique was used in selecting the study communities. At the individual level, random sampling technique was applied, and 461 women were recruited from 21^st^ February 2021–21^st^ March 2021. Using a questionnaire, a face-to-face approach was used to conduct the interviews. The questionnaire included questions on socio-demographic characteristics, place of childbirth and factors that led to the choice of delivery place. Using Statistical Package for Social Sciences version 25, descriptive and binary logistic regression analysis were conducted.

**Results:**

The results revealed that 64.0% of the women delivered in health facilities during the pandemic. Health facility delivery was more likely to occur among women with higher educational status (AOR: 5.2; 95% CI: 1.40–19.40), married women (AOR:6.3; 95% C.I:1.10–35.80), active National Health Insurance Scheme holders during delivery (AOR: 13.8; 95% C.I: 4.60–41.90), women who received education on birth preparedness and complication readiness (AOR: 7.6; 95% C.I:3.30–17.50) and women with underlying conditions before pregnancy (AOR:3.3; 95% C.I:1.20–9.20). There were reduced odds of health facility delivery among women with a history of home delivery (AOR:0.2; 95% C.I:0.10–0.50), when the mother-in-law decides on the place of delivery (AOR:0.1; 95% C.I:0.03–0.50), longer distance to the place of delivery (AOR:0.3; 95% C.I:0.01–1.00) and when women perceived COVID-19 as a barrier to facility delivery (AOR:0.1; 95% C.I:0.03–0.20).

**Conclusion:**

Our findings show that health facility delivery declined during COVID-19. Factors that affected health facility delivery were educational status, marriage, having an active National Health Insurance Scheme, education on birth preparedness and complication readiness, underlying conditions before pregnancy, history of home delivery, mother-in-law decision on place of delivery, distance to place of delivery and perceiving COVID-19 as a barrier to facility delivery. These contributed to low facility delivery. Thus, maternal health services need to be brought to the doorsteps of communities, including proper implementation of the Focused Antenatal Care and community-based pregnancy school programmes, especially during future pandemics.

## Introduction

The coronavirus disease 2019 (COVID-19) was first designated as an international public health emergency by the World Health Organisation (WHO) in January 2020 [1]. In March 2020, it escalated to a global pandemic [1–3]. WHO-recommended public health and social measures, like banning both private and public gatherings, were implemented in the world to prevent the spread of COVID-19 [4,5]. Following the WHO’s recommendations, the Ghanaian government enacted measures like mandatory border closures, increased community surveillance, mandatory quarantine for all foreign visitors, and mandatory self-quarantine for peoples who had close contact with a sick person or suspected case [6,7].

Adverse pregnancy outcomes, high incidence of hospitalisation and maternal mortality were prevalent during previous epidemics such as the severe Acute Respiratory Syndrome (SARS) and Middle East respiratory disease [8–10]. Similarly, the COVID-19 pandemic had a variety of effects on the use of health services. Prenatal care attendance decreased in several health facilities as a result of lockdowns, financial hardships, and concerns about SARS-CoV-2 infection [6,11,12]. During this period, Ghana’s healthcare system experienced disruptions in a number of areas, including maternal health services [13,14]. Infrastructure, healthcare staff, supply chains, and budgetary constraints limited health systems’ capacity to provide services and make quick adjustments [15]. Pregnant women’s access to healthcare was altered as a result of physical distance and infection control regulations, including fewer in-person appointments, a rise in telehealth use, and restrictions on the availability of support services during pregnancy [16].

The accessibility of basic health services was impacted by shortened clinic hours, patient intake limits, and changes in the types of services provided [17–19]. This was due to the limited resources to respond to the pandemic. Additionally, there was a need to reallocate resources away from routine health activities. Health service accessibility and care-seeking of clients were also hampered by demand-side issues such as mobility limitations, public transportation shutdowns, perceived changes in service quality, and anxiety about COVID-19 infection at health facilities [20,21].

Some studies have demonstrated how COVID-19 made pre-existing gender and social class disparities worse, with substantial evidence indicating that the pandemic disproportionately affected women and lower-income populations [22–25]. Broader social and structural factors may have also constrained women’s choices during the pandemic, with women often lacking decision-making power on the place to deliver a baby [26–28]. In Ghana, healthcare institutions and some Non-Governmental Organisations, over the years, have worked tremendously to improve facility delivery that aims to reduce newborn, child and maternal mortality [29]. Despite these efforts, health facility delivery and maternal mortality remain high (106 deaths per 100,000 live births), with a higher burden of maternal mortality in the Northern Region [30,31]. The prevalence of health facility delivery reduced from 65% in 2014 to 56.8% in 2015 and further decreased to 54.6% in 2019, just before the COVID-19 outbreak [32–34]. Other studies reported on some factors that hinder skilled delivery, such as healthcare professionals’ attitudes and the educational level of expectant mothers [29,35]. In 2021, the Ministry of Health in Ghana reported that health facility delivery had increased by 5.4 percentage points to 63.5% [36], while Asumadu et al. also reported a health facility delivery of 72.4% during the pandemic [6]. However, these studies failed to report on the factors that could have accounted for the increment in health facility delivery in the face of the pandemic. Ayanore et al., who reported on the socio-economic determinants of general healthcare utilisation during the pandemic, without an explicit focus on marternal healthcare services particulary facility-based childbirth [37]. This study, therefore, assessed the prevalence and associated factors of facility delivery during COVID-19 in the Tamale Metropolis of Ghana.

## Materials and methods

### Study design and setting

An analytical cross-sectional design was implemented in the Tamale Metropolis of Ghana. This design measures the prevalence of health outcomes, determinants of health, and describe features of the population [38]. The population of Tamale Metropolis, as reported in the 2021 Population and Housing Census (PNC), was 374,744, with 185,051 and 189,693 being males and females respectively [39]. The Metropolis has a total landmass of 646.901.80 sq km [39]. The Northern Region of Ghana has 16 administrative districts, including the Tamale Metropolis, which is located in Tamale, the region’s headquarters. Tamale North Sub-Metropolitan, Tamale Central Sub-Metropolitan, and Tamale South Sub-Metropolitan areas are the three sub-metros that make up the metropolitan area. There are four major public hospitals in the Tamale Metropolis comprising; Tamale Teaching Hospital (TTH), Tamale Central Hospital (TCH), Tamale West Hospital (TWH) and Tamale Seventh Day Adventist (SDA) Hospital. The Tamale Teaching Hospital serves as a tertiary referral centre for all health facilities in northern Ghana [40,41].

### Population and sampling

The target population was mothers who delivered during the pandemic and permanently lived in the Tamale Metropolis. Women who had given birth before March 2020 were excluded from the data collection. For mothers with multiple children, the researchers considered the last birth and whether the delivery occurred after March 2020. Additionally, women who were sick or mentally unwell were excluded from the study. The data collection spanned from 21^st^ February 2021–21^st^ March 2021.

The sample size for the study was determined using Fisher’s et al (1998) formula:


$$n=\frac{Z^{\text{2}}\text{ P }(\text{1}\;-\text{ P})}{e^{\text{2}}}$$

Where, n = sample size

Z = the value for the corresponding confidence level (that is, 1.96 for a 95% confidence level)

P = the estimated value for the proportion of the target population of interest is 50%

e = the level of statistical significance set, which is 5% with a 95% confidence level [42]


$$n=\frac{{\text{1}.\text{9}\text{6}}^{\text{2}}\text{ x }\text{0}.\text{50 }(\text{1}\;-\;\text{0}.\text{50})\;}{{\text{0}.\text{0}\text{5}}^{\text{2}}}$$

$$=\frac{\text{3}.\text{84 x }\text{0}.\text{50 x }\text{0}.\text{05}}{\text{0}.\text{0025}}$$

= 384

The sample size for this study was 384 respondents. About 20% for non-responses was added to the sample size, making a total of 461. A high proportion was added because the researchers anticipated a low response rate due to the COVID-19 pandemic.

The researchers adopted a multistage sampling technique. In the first stage, the Tamale Metropolis was categorised into five clusters: Tamale North, South, East, West and Central. Each of these clusters shared similar characteristics including sociodemographic information and health seeking behaviour [39]. Three clusters were selected randomly through the balloting technique. Names of all five clusters were written on pieces of paper, folded by one research assistant and placed in an opaque box. A second research assistant was asked to mix the papers thoroughly, and a third selected three papers. This resulted in the selection of the North, South, and Central clusters. In the second stage, the names of all the communities in each of the three clusters were written on pieces of paper again, folded and placed inside an opaque box, and one community selected randomly. This was also conducted by three research assistants. This approach was repeated for the three clusters. Thus, Kalpohin was selected for the North, Sakasaka for the Central and Vittin for the South clusters. Using the ratio and proportion method (probability proportional to size sampling), the number of respondents to be recruited from each cluster was determined. This resulted in the following quotas: 128 for Kalpohin, 141 for Sakasaka and 192 for Vittin. In each of the selected communities, a random technique was applied. This technique was achieved by identifying the centre of a selected community and spinning a pen. The research team followed the direction of the pen and interviewed every 2^nd^ qualified person as respondents at their household levels. Each household had a chance for only one respondent, regardless of the number of women who met the inclusion criteria. When there was more than one woman in a household, a respondent was decided randomly by the toss of a coin.

### Data collection tools and procedure

The data was gathered through a questionnaire developed by the research team. The questionnaire was created after evaluating prior research literature. To confirm the correctness and appropriateness of the questions, the questionnaire was face-validated through a mutual consensus process involving multiple consultations among the authors. Three of the authors are subject experts; two are registered nurses and two PhD holders with extensive teaching and research experience in academic sectors. The questionnaire was pre-tested in Zugbeli, a community within Tamale West with similar socio-demographic characteristics to the study area. Research assistants fluent in the Dagbani language (main local language of inhabitants of Tamale Metropolis) were recruited and trained. Face-to-face approach was used to administer the questionnaires at the respondents’ homes. Respondents were allowed to choose either the Dagbani language or English language as their preferred medium of communication. The trained research assistants administered the questionnaires directly. The questionnaire had two sections; section A focused on the socio-demographic characteristics of the respondents and section B asked questions about antennal care (ANC) attendance, place of delivery, history of home delivery, National Health Insurance Scheme (NHIS) status, person who decides on a place of delivery, distance to the nearest health facility, whether respondents were educated on birth preparedness and complication readiness, women perceiving COVID-19 as a barrier to facility delivery and any underlying condition before pregnancy. Each interview lasted about 30 minutes.

### Data processing and analysis

The data were entered and stored in the Statistical Package for Social Sciences (SPSS) version 25 software. The results were presented as descriptive statistics including percentages and frequencies. Binary logistic regression was used to assess the associations between the dependent (choice of place of delivery) and independent variables at a confidence level of 95% and 5% alpha (α) or a p-value. The independent variables were selected based on evidence from previous studies and contextual realities. Age, education, marital status, place of residence and history of home delivery have been reported to be associated with the choice of a place of delivery [35,43]. Other factors, such as active NHIS during delivery, distance to place of delivery, education on birth preparedness and complication readiness and underlying medical condition before pregnancy reported [44–47] were included in the analysis to test their association with the place of delivery during the pandemic. In some northern parts of Ghana, the head of the family has more authority in deciding the family’s health-seeking behaviour, including maternal health services [48]. Therefore, it was essential for this study to include those who decide about the place of birth in the analysis including the husband/partners and mother-in-law. The dependent variable was coded as ‘1’ for facility delivery and ‘0’ for home delivery. An unadjusted (univariate) binary logistic regression model was constructed for each independent variable to generate crude odds on its association with the dependent variable. To control for confounders, a multivariate binary logistic regression model was built to measure the association between the dependent and independent variables [49].

### Ethics considerations

Ethics approval for this study was received from the Kwame Nkrumah University of Science and Technology (KNUST) Committee on Human Research and Publication Ethics (CHRPE/AP/494/20). The respondents were informed of the possible benefits of the study as well as the risks and discomfort involved. Furthermore, respondents were informed that their participation in the research was voluntary, and they could withdraw from the study at any time without consequences. Written informed consent was obtained from each respondent before commencement of the study. The informed consent form was read to the respondents in their preferred language and they were made to sign or thumbprint on the form to indicate their agreement. The research assistants and respondents also adhered to the COVID-19 safety protocols throughout the field survey.

## Result

### Socio-demographic characteristics and health service factors

A total of 450 out of 461 respondents participated in this study, representing a 98% response rate (Table 1). The result shows 51.8% of the respondents were aged 26–35 years. More than half of the respondents (52.2%) had no formal education. Most of the women (94.9%) were married. It was found that more than half (52.0%) of the women were unemployed, and 68.9% of them live in an urban area. The proportion of women who did not attend ANC was 24.4%. The analysis showed that 36.0% of the women delivered at home, and 38.2% had a history of home delivery. Women with no active NHIS during delivery accounted for 23.3%. When the women were asked about who decides about the place of delivery, 19.6% said their husbands, while 7.3% said their mothers-in-law. According to 27.3% of the women, the distance between their home and the nearest place of delivery was about 11–30 km. Women who did not receive education on birth preparedness and complication readiness accounted for 28.9%. The analysis showed 33.8% of women perceived COVID-19 as a barrier to delivering at a health facility, while 14.2% had an underlying condition before pregnancy.

**Table 1:** Socio-demographic characteristics and health service factors.

Socio-demographic characteristics	Frequency	Percentage (%)
**Age**		
<=25	164	36.4
26-35	233	51.8
>=36	53	11.8
**Level of education of respondents**		
No education	235	52.2
Primary	48	10.7
Junior High School	85	18.9
Senior High School	82	18.2
**Marital status**		
Single	23	5.1
Married	427	94.9
**Occupation**		
Farmer	30	6.7
Trader	134	29.8
Unemployed	234	52.0
Public servant	22	4.9
Others specify	30	6.7
**Area of residence**		
Urban	310	68.9
Rural	140	31.1
**ANC attendance**		
Yes	340	75.6
No	110	24.4
**History of home delivery**		
Yes	172	38.2
No	278	61.8
**On active NHIS during delivery**		
Yes	345	76.7
No	105	23.3
**Who decided on choosing a place of delivery?**		
Myself	238	52.9
Husband	88	19.6
Mother-in-law	33	7.3
Myself and my husband	91	20.2
**Distance between respondents’ houses and the nearest place of delivery**		
<= 10 km	327	72.7
11–30 km	123	27.3
**Education received on birth preparedness and complication readiness**		
Yes	320	71.1
No	130	28.9
**COVID-19 as a perceived barrier to delivering at a health facility**		
Yes	152	33.8
No	298	66.2
**Any underlying condition before pregnancy**		
Yes	64	14.2
No	386	85.8

*ANC-Antenatal care; NHIS-National Health Insurance Scheme*

### Prevalence and factors associated with health facility delivery during COVID-19

The prevalence of health facility-based delivery was 64.0% (n = 288), and that of home-based delivery was 36% (n = 162). Table 2 presents the results of the factors associated with facility-based delivery during COVID-19. The results show that compared to home delivery, women with senior high school education were statistically significantly more likely to deliver at the health facility than women with no education (AOR = 5.2; 95%CI: 1.40–19.40).

**Table 2:** Factors associated with health facility delivery during COVID-19.

Variables	Health facility delivery (n=288)		Home delivery (n=162)		COR (95% C.I)	P-value	AOR (95% C.I)	P-value
	Freq	%	Freq	%				
**Age**								
<=25 years	98	34.0	66	40.7	Ref		Ref	
26-35 years	163	56.6	70	43.3	1.5 (1.00-2.40)	0.035	2.3 (1.00-5.40)	0.062
>=36 years	27	9.4	26	16.0	0.7 (0.40-1.30)	0.260	2.8 (0.70-10.30)	0.143
**Education status**								
No education	131	45.5	104	64.2	Ref		Ref	
Primary	30	10.4	18	11.1	1.3 (0.70-2.50)	0.390	2.8 (0.70-10.60)	0.128
Junior High School	60	20.8	25	15.4	1.9 (1.10-3.20)	0.018	1.5 (0.60-4.00)	0.401
Senior High School	67	23.3	15	9.3	3.5 (1.90-6.60)	<0.001	5.2 (1.40-19.40)	0.014
**Marital status**								
Single	8	2.7	15	9.3	Ref		Ref	
Married	280	97.2	147	90.7	3.6 (1.50-8.60)	0.005	6.3 (1.10-35.80)	0.039
**Occupation**								
Unemployed	144	50.0	90	55.6	Ref		Ref	
Farmer	7	2.4	23	14.2	9.7 (3.80-24.40)	<0.001	3.9 (0.60-23.10)	0.139
Trader	100	34.7	34	21.0	5.3 (2.20-12.80)	<0.001	1.9 (0.30-10.20)	0.481
Public servant	17	10.5	5	3.1	11.2 (3.00-41.30)	<0.001	2.2 (0.20-23.80)	0.512
Others	10	3.5	10	6.2	6.6 (2.10-20.50)	<0.001	3.9 (0.30-53.20)	0.313
**Residence**								
Urban	215	69.4	95	30.6	Ref		Ref	
Rural	73	52.1	67	47.9	0.5 (0.30-0.70)	<0.001	1.6 (0.60-4.30)	0.374
**History of home delivery**								
No	208	72.2	70	43.2	Ref		Ref	
Yes	80	27.8	92	56.8	0.3 (0.20-0.40)	<0.001	0.2 (0.10-0.50)	0.0010
**Active NHIS during delivery**								
No	10	3.5	95	58.6	Ref		Ref	
Yes	278	96.5	67	41.4	39.4 (19.50-79.70)	<0.001	13.8 (4.60-41.90)	<0.001
**The person deciding on the place of delivery**								
Myself	192	66.7	46	28.4	Ref		Ref	
Husband	56	19.4	32	19.8	0.4 (0.20-0.70)	0.002	1.0 (0.40-2.70)	0.990
Mother-in-law	6	2.1	27	16.7	0.1 (0.02-0.10)	<0.001	0.1 (0.03-0.5)	0.005
Husband and I	34	11.8	57	35.2	0.1 (0.10-0.20)	<0.001	1.4 (0.40-4.70)	0.567
**Distance to place of delivery**								
<= 10 km	260	90.3	67	41.4	Ref		Ref	
11-30 km	28	9.7	95	58.6	0.1 (0.05-0.10)	<0.001	0.3 (0.10-1.00)	0.041
**Education on birth preparedness and complication readiness**								
No	27	9.4	103	63.6	Ref		Ref	
Yes	261	90.6	59	36.4	16.9 (10.10-28.10)	<0.001	7.6 (3.30-17.50)	<0.001
**COVID-19 as a perceived barrier to delivering at a health facility**								
No	233	80.9	153	94.4	Ref		Ref	
Yes	33	11.5	119	73.5	0.05 (0.03-0.01)	<0.001	0.1 (0.03-0.20)	<0.001
**Any medical condition before pregnancy**								
No	233	80.9	153	94.4	Ref		Ref	
Yes	55	19.1	9	5.6	4.0 (1.90-8.40)	<0.001	3.3 (1.20-9.20)	0.024

*ANC-Antenatal Care; NHIS-National Health Insurance Scheme;* COR: Crude Odds Ratio; C.I: Confidence Intervals; AOR: Adjusted Odds Ratio.

Nearly all (97.2%) married women delivered at a health facility during the pandemic. The likelihood for a married woman to opt for health facility delivery was high (AOR = 6.3; 95%CI: 1.10–35.80) compared to the unmarried mothers. Over a quarter (27.8%) of the women with a history of home delivery gave birth at a health facility. This depicts a reduced odds ratio of health facility delivery among such mothers (AOR = 0.2; 95%CI: 0.10–0.50). The majority of the women (96.5%) with active NHIS gave birth at a health facility during the COVID-19 pandemic. This further showed that women with active NHIS during delivery were more likely to deliver at the health facility (AOR = 13.8; 95%CI: 4.60–41.90) than those without active NHIS. The analysis revealed reduced odds ratio for health facility delivery of respondents whose mother-in-law decides on the place of delivery (AOR = 0.1; 95%CI: 0.03–0.50). That is, 2.1% of such respondents delivered at the health facility during the COVID-19 pandemic. A few (9.7%) of the women who had to travel 11–30 km to the nearest health facility opted for facility delivery with a reduced odds of AOR = 0.3; 95%CI: 0.10–1.00. There were significantly higher odds ratio of facility delivery among women who received education on birth preparedness and complication readiness (AOR = 7.6; 95%CI: 3.30–17.50). The results revealed that 90.6% of these women delivered at a health facility during the COVID-19 pandemic. Women who perceived COVID-19 as a barrier to delivering at a health facility showed reduced odds ratio and prevalence of health facility delivery presented as (AOR = 0.1; 95%CI: 0.03–0.20) and 11.5% respectively. For women with any medical condition before pregnancy, 19.1% gave birth at a health facility during the pandemic with a reduced odds ratio of (AOR = 3.3; 95%CI: 1.20–9.20).

## Discussion

Our study assessed factors associated with health facility delivery during COVID-19 among mothers in the Tamale Metropolis of Ghana.

Before the pandemic, studies had reported a significant increase in health facility delivery. For instance, Asumadu et al., [6] compared health facility delivery before and during COVID-19 and reported 76.8% and 72.4%, respectively. Similarly, our study found a reduced health facility delivery of 64.0% during the COVID-19 pandemic. This finding is similar to a study that also reported a decline in maternal health services in Ghana [50].

The educational level of women was found to be directly associated with health facility delivery. Thus, respondents who are educated up to the secondary school level had a higher chance of delivery at the health facility. Similarly, previous studies have reported that the choice of health facility for delivery was associated with the level of education attained by women [35,51–53]. The reason could be that, women with higher levels of education have greater awareness of the risks associated with pregnancy and unskilled birth, therefore more inclined to give birth at a health facility. Furthermore, educated women have greater autonomy and are more concerned about their health and wellbeing. Thus, they are empowered to make health-related decisions, which ultimately enhances their behaviour when seeking healthcare [54]. The educated women may put in additional efforts against the odds despite fear of the pandemic to adhere to the COVID-19 protocols to access essential services like health facility delivery as compared to mothers with no formal education who may not have the ability to make informed decisions.

In this study, the findings revealed that married women were 6 times more likely to choose health facility delivery than single women. It also revealed that women who chose a place of delivery based on their mother-in-law’s influence were less likely to opt for health facility delivery. This implies that women living with their spouse may have autonomy over their maternal health and may choose health facility delivery over home birth. The finding is supported by studies from Indonesia that reported that women who live in their own homes are more likely to deliver at healthcare facilities [55,56]. Studies have also shown that cultural norms play a significant role in influencing women to give birth at home and to seek the services of traditional birth attendants [57,58]. Some could argue that mothers-in-law may cling to the culture about the place of delivery and impose this on their daughters-in-law. Thus, expectant mothers staying with their mothers-in-law may have less likelihood of health facility delivery. Additionally to the cultural influence from mothers-in-law on expectant mothers to opt for home delivery, these in-laws could have also used the COVID-19 pandemic as a rationale to discourage the pregnant women from health facility delivery.

In this study, women who had to travel 11–30 km of distance during the COVID-19 pandemic to access a place of delivery were less likely to practice health facility delivery. Before the pandemic, studies had reported how geographical location and distance affect health facility delivery [45,46,59]. However, the COVID-19 pandemic could have made maternal health services more difficult for expectant mothers. As the restrictions during the pandemic affected almost all aspects of life, health facility delivery was equally impacted. The distance to access skilled delivery services might have influenced the prevalence of health facility delivery. For instance, a study conducted in Kenya revealed that women who gave birth at home during the COVID-19 pandemic did so due to the inaccessibility of health facilities, such as transportation [60]. This implies that as economic activities reduced during the pandemic, including public transport, mothers were forced to opt for home delivery rather than health facility delivery.

In this study, it was found that women without active NHIS membership were less likely to practice health facility delivery. This confirms the earlier assertion that urban and poor women were more likely to deliver at home, especially those without active NHIS. Aside from the economic hardship resulting from COVID-19, the pandemic interrupted some services such as the renewal of the NHIS membership. This could influence the process of choosing a place of delivery. A study conducted in Ghana reported that the impact of NHIS on the use of delivery services by various health service providers varied. Being insured raises the likelihood of using health facilities for delivery in comparison to home delivery [44]. Women with history of home delivery were less likely to opt for facility delivery. Our findings support other studies that previous health facility delivery is associated with higher odds of delivering at a health facility [35,46]. These women may have chosen to give birth at home because they perceived a trouble-free home delivery experience in the past and expected to be in a familiar situation where they could feel soothed by their loved ones nearby. Additionally, they could justify their assertion that the presence of COVID-19 makes health facility delivery unsafe due to the chance of transmission.

Our findings depicted that women who did not receive education on birth preparedness and complication readiness were less likely to deliver in a health facility. This could be because the women were not aware of the dangers or complications associated with unskilled delivery. However, the factors accounting for these women not receiving the education could be traced to the COVID-19 pandemic. A study reported that women who are well prepared for childbirth have a greater likelihood of going for institutional deliveries than women with no preparation [47]. However, the restrictions imposed on people to curb the spread of the virus could deter mothers from receiving essential healthcare services.

Women who considered COVID-19 a perceived barrier to health facility delivery were less likely to give birth at a health facility. This was a result of the restrictions put in place and the fear of infections from health facility delivery.

## Strengths and limitations

There are strengths to this study. The communities in the Tamale Metropolis had an equal chance of being chosen according to a multistage sampling technique. Additionally, the spread of the community selection reflects a representative sample of the study population. To reduce bias in the selection of respondents, a random sample technique was used. The study is also community-based, and respondents completed questionnaires in familiar environments. This study does, however, have certain drawbacks. The adoption of ‘spinning a pen’ method to initiate sampling may introduce bias in participant selection. The findings are also susceptible to recall bias. The reliability of the information was predicated on the respondents’ capacity for accurate memory. The study was conducted in three selected communities within one metropolis, which restricts the geographic representativeness of the findings. Some of the findings are rooted in culture and social context, and reproducibility in different cultures might be difficult.

## Conclusion

Our findings show that health facility delivery declined during the COVID-19. There was a link between health facility delivery and other factors including maternal education, marital status, active National Health Insurance Scheme membership during delivery, receiving education on birth preparedness and complication readiness during the COVID-19 period, having underlying conditions before pregnancy, history of home delivery, mother-in-law decision on place of delivery, distance to place of delivery and perceiving COVID-19 as a barrier to facility delivery. Thus, expectant mothers need to be able to automatically renew their health insurance membership. Women with a history of home delivery and those living with mothers-in-law should be given further attention, particularly, Focused Antenatal Care programme by the Ghana Health Service. There is a need to prioritize preparation and use of essential maternal health services during pandemics.

## Supporting information

S1 File S1 21022026. (XLSX)
